# Investigation of the influence of high glucose on molecular and genetic responses: an *in vitro* study using a human intestine model

**DOI:** 10.1186/s12263-018-0602-x

**Published:** 2018-04-30

**Authors:** Tugce Boztepe, Sukru Gulec

**Affiliations:** Molecular Nutrition and Human Physiology Laboratory, Food Engineering Department, İzmir Institute of Technology, Room: 215, Urla, 35430 İzmir, Turkey

**Keywords:** Obesity, Intestine, High glucose consumption, Caco-2, *ABCA1*, *IRX3*, *TXNIP*, *LCN15*

## Abstract

**Background:**

Dietary glucose consumption has increased worldwide. Long-term high glucose intake contributes to the development of obesity and type 2 diabetes mellitus (T2DM). Obese people tend to eat glucose-containing foods, which can lead to an addiction to glucose, increased glucose levels in the blood and intestine lumen, and exposure of intestinal enterocytes to high dietary glucose. Recent studies have documented a role for enterocytes in glucose sensing. However, the molecular and genetic relationship between high glucose levels and intestinal enterocytes has not been determined. We aimed to identify relevant target genes and molecular pathways regulated by high glucose in a well-established in vitro epithelial cell culture model of the human intestinal system (Caco-2 cells).

**Methods:**

Cells were grown in a medium containing 5.5 and 25 mM glucose in a bicameral culture system for 21 days to mimic the human intestine. Transepithelial electrical resistance was used to control monolayer formation and polarization of the cells. Total RNA was isolated, and genome-wide mRNA expression profiles were determined. Molecular pathways were analyzed using the DAVID bioinformatics program. Gene expression levels were confirmed by quantitative reverse transcription polymerase chain reaction (RT-qPCR).

**Results:**

Microarray gene expression data demonstrated that 679 genes (297 upregulated, 382 downregulated) were affected by high glucose treatment. Bioinformatics analysis indicated that intracellular protein export (*p* = 0.0069) and ubiquitin-mediated proteolysis (*p* = 0.024) pathways were induced, whereas glycolysis/gluconeogenesis (*p* < 0.0001), pentose phosphate (*p* = 0.0043), and fructose-mannose metabolism (*p* = 0.013) pathways were downregulated, in response to high glucose. Microarray analysis of gene expression showed that high glucose significantly induced mRNA expression levels of thioredoxin-interacting protein (*TXNIP*, *p* = 0.0001) and lipocalin 15 (*LCN15*, *p* = 0.0016) and reduced those of ATP-binding cassette, sub-family A member 1 (*ABCA1*, *p* = 0.0004), and iroquois homeobox 3 (*IRX3*, *p* = 0.0001).

**Conclusions:**

To our knowledge, this is the first investigation of high glucose-regulated molecular responses in an intestinal enterocyte model. Our findings identify new target genes that may be important in the intestinal glucose absorption and metabolism during high glucose consumption.

**Electronic supplementary material:**

The online version of this article (10.1186/s12263-018-0602-x) contains supplementary material, which is available to authorized users.

## Background

Consumption of high dietary glucose is a risk factor for the development of obesity and type 2 diabetes mellitus (T2DM) in humans [1, 2]. The total number of deaths attributable to high blood glucose in 2012 was estimated as 3.7 million, 1.5 million of which were due to diabetes [3]. High glucose intake leads to postprandial glycemia, insulinemia, and lipidemia in healthy overweight people [4]. Moreover, longer exposure to dietary high glucose is associated with the development of T2DM [5]. These findings indicate that control of dietary glucose intake is an important strategy to combat the development of obesity and T2DM. Recent studies have focused on the mechanisms controlling blood glucose turnover during obesity and T2DM. For example, sodium-dependent glucose transporter 1 (SGLT1) is a protein that has been targeted to control reabsorption of glucose from the kidney [6]. Moreover, hepatic glucose production from glycogen has been studied as a therapeutic target molecular pathway in the development of T2DM [7]. Glucose-dependent molecular mechanisms contributing to obesity and T2DM have been extensively studied in the adipose tissue, liver, pancreas, and brain. The small intestine may also be an important tissue to investigate glucose metabolism during obesity and T2DM, as it can contribute to systemic glucose production in fasted and diabetic states [8]. Moreover, the gene expression and morphology of the small intestine are altered during obesity [9]. These adaptive changes of the small intestine may be uncontrolled during high nutrient consumption and metabolic disease states. The small intestine mainly consists of enterocytes, which directly interact with dietary nutrients and transfer them into the circulatory system [10]. The rate of interaction between nutrients and enterocytes varies depending on the eating habits of individuals.

Recent studies suggest that there may be a physiological interaction between enterocytes and glucose that contributes to intestinal dietary glucose sensing [9, 11, 12]. In humans, high dietary glucose consumption elevates glucose levels in the lumen of the intestine and in the blood; hence, enterocytes are exposed to high levels of glucose in both the diet and the blood. This situation can be continuous in obese people, due to the consumption of foods containing high levels of glucose [13]. It is unknown how enterocytes respond to high glucose levels in either the diet or the blood. Hence, investigations to improve the understanding of the adaptive physiological changes of enterocytes in response to high glucose consumption are warranted. In this study, we examined the genome-wide molecular responses of enterocytes to high glucose. Our results demonstrate that chronic high glucose exposure influences specific molecular pathways and genes that may be important in the control of intestinal glucose absorption during high glucose consumption.

## Results

### Effect of high glucose on cellular tight junctions and measurement of glucose efflux

The monolayer structure formation and tight junction permeability of Caco-2 cells are commonly evaluated by measurement of transepithelial electrical resistance (TEER) along with *OCLN* and *ZO1* gene mRNA levels [14, 15]. We grew Caco-2 cells in bicameral cell culture chambers to mimic the human intestine and measured TEER and *OCLN* and *ZO1* mRNA expression levels to evaluate the intestinal barrier function of our experimental model. The TEER levels of control and high glucose-treated 21-day postconfluent Caco-2 cell groups were not significantly different (Table 1) and neither were *OCLN* and *ZO1* mRNA levels significantly affected by high glucose treatment (Table 1). These results demonstrate that high glucose did not interfere with barrier formation by monolayer Caco-2 cells.

**Table 1:** Effect of high glucose on cell permeability

	Ctrl	+Glc	*p* value	Significance
TEER (ohm/cm^2^)	934 ± 32.35	809.5 ± 39.29	0.0707	NS
Normalized *OCLN* mRNA levels	1 ± 0.095	0.93 ± 0.10	0.6096	NS
Normalized *ZO1* mRNA levels	1 ± 0.064	1.05 ± 0.14	0.7336	NS

Caco-2 cells were grown on collagen-coated inserts in 5.5 and 25 mM glucose containing DMEMs for 21 days. TEER value was measured before RNA isolation. Occludin (*OCLN*) and Zoolin1 (*ZO1*) mRNA levels were measured by RT-qPCR, and relative fold changes of gene expression levels were analyzed. Statistical significance was determined by Student’s *t* test. Values are expressed as the mean ± SD; *n* = 3 independent experiments with three technical replicates

The function of enterocytes involves absorption of dietary nutrients (nutrient uptake) and their transport into the blood circulation (nutrient efflux). We tested cellular glucose efflux under control and high glucose treatment conditions. Glucose efflux was significantly increased in the glucose-treated group relative to the controls at the 90-min (*p* = 0.0031; 1.4-fold), 120-min (*p* = 0.01; 1.4-fold), and 240-min (*p* = 0.0049; 1.9-fold) time points (Table 2), suggesting that Caco-2 cells mimic the human intestine in terms of glucose efflux in our experimental model.

**Table 2:** Cellular glucose efflux

Time (min)	Ctrl	+Glc	*p* value	Significance
0	105.5 ± 4.50	114.5 ± 2.5	0.2225	NS
30	104.5 ± 1.50	121.0 ± 7.0	0.1477	NS
60	103.5 ± 5.50	128.0 ± 2.0	0.0526	NS
90	96.5 ± 2.50	135.5 ± 2.5	0.0031	Yes
120	105.5 ± 4.00	142.5 ± 3.5	0.0195	Yes
240	90.00 ± 5.0	169.5 ± 2.5	0.0049	Yes

The glucose efflux was measured from the basolateral surface of the two experimental groups at 0-, 30-, 60-, 120-, 180-, and 240-min time points. Results were given as milligrams per deciliter. Statistical significance was determined by Student’s *t* test. Values are expressed as the mean ± SD; *n* = 3 independent experiments with three technical replicates

### High glucose alters mRNA expression levels of glucose-regulated genes

Before genome-wide array analysis was performed, we tested whether high glucose affected the regulation of glucose-related genes in Caco-2 cells. Thus, we analyzed mRNA expression of *GLUT2*, *GLUT5*, and *SGLT1*. mRNA expression levels of *GLUT2* (1.8-fold; *p* = 0.0001), *GLUT5* (1.5-fold; *p* = 0.0004), and *SGLT1* (1.4-fold; *p* = 0.0053) were significantly lower in the high glucose-treated group relative to the controls (Table 3).

**Table 3:** mRNA expression levels of glucose-regulated genes

Genes	Ctrl	+Glc	*p* value	Significance
*GLUT2*	1.004 ± 0.035	0.5465 ± 0.055	0.0001	Yes
*GLUT5*	1.014 ± 0.064	0.6490 ± 0.044	0.0004	Yes
*SGLT1*	1.023 ± 0.083	0.7186 ± 0.040	0.0053	Yes

Total RNA was isolated from the control and the high glucose-treated (+Glc) groups. RT-qPCR was performed to analyze relative expression levels of genes from the two experimental groups. Statistical significance was determined by Student’s *t* test. Values are expressed as the mean ± SD; *n* = 3 independent experiments with three technical replicates

### Classification of differentially expressed genes and confirmation of mRNA expression of genes from molecular pathways

Microarray technology was used to identify genes that were differentially expressed in experimental samples. When we compared the control and high glucose-treated groups, we identified a total of 297 and 382 genes that were significantly up- and downregulated, respectively (≥ 1.5-fold change; *p* < 0.05) in response to high glucose treatment. The list of high glucose-regulated genes is available under GEO accession number GSE97977 ( https://www.ncbi.nlm.nih.gov/geo/query/acc.cgi?acc=GSE97977; Additional file 2: Table S2 and Additional file 3: Table S3). These genes were used to identify high glucose-regulated molecular pathways by DAVID bioinformatics program using KEGG Pathways database. The gene IDs were converted to DAVID gene IDs that were associated with particular pathway annotation terms. The enrichment of molecular pathways was performed by Benjamini-Hochberg method, and *p* ≤ 0.05 was used as a cutoff value in DAVID annotation model. Intracellular protein export (*p* = 0.0069) and ubiquitin-mediated proteolysis (*p* = 0.024) pathways were identified as significantly induced, whereas glycolysis-gluconeogenesis (*p* < 0.0001), pentose phosphate (*p* = 0.0043), and fructose-mannose metabolism (*p* = 0.013) pathways were significantly downregulated by high glucose treatment in Caco-2 cell monolayers after 21 days (Table 4). The detailed information for high glucose-regulated pathways can be found in Additional file 4: Figure S2, Additional file 5: Figure S2, Additional file 6: Figure S3, Additional file 7: Figure S4, and Additional file 8: Figure S5.

**Table 4:** Functional analysis of high glucose-regulated up- or downregulated genes

Upregulated pathways	*p* value	Number of genes
Ubiquitin-mediated proteolysis	2.4 × 10^−2^	7
Protein export	6.9 × 10^−3^	3
Downregulated pathways		
Glycolysis/gluconeogenesis	3.0 × 10^−5^	10
Pentose phosphate	4.3 × 10^−3^	5
Fructose and mannose metabolism	1.3 × 10^−2^	5

Microarray data showed a total of 297 upregulated and 382 downregulated genes in response to high glucose treatment. The high glucose-regulated genes were subjected in DAVID bioinformatics analyze program to describe the functional molecular pathways along with the number of genes in each pathway

### Quantitative reverse transcription polymerase chain reaction analysis of genes identified by microarray analysis

Genome-wide mRNA array technology provides valuable information about the genomic regulation of cells; however, technical procedures can affect microarray results [16]. Therefore, we performed RT-qPCR experiments to confirm the regulation of individual genes from the identified molecular pathways. In the intracellular protein export pathway, mRNA levels of *SRP14* (1.4-fold; *p* = 0.002) and *SRP54* (1.8*-*fold; *p* = 0.0003) and in the ubiquitin-mediated proteolysis pathway, mRNA levels of *UBE2L6* (2-fold; *p* = 0.0007), *UBE2Q2* (1.6-fold; *p* = 0.01), and *SKP1* (1.5-fold; *p* = 0.03) were significantly increased in the high glucose-treated group compared with the controls (Table 5). However, RT-qPCR analysis of the *SRP72* gene in the protein export pathway did not indicate similar regulation to that determined by microarray analysis. We also selected common genes from molecular pathways that were downregulated by high glucose treatment and found that mRNA levels of *ALDOA* (1.5-fold; *p* = 0.006), *ALDH2* (2.8-fold; *p* = 0.01), *PFKP* (2.9-fold; *p* = 0.0002), *PGAM1* (1.7-fold; *p* = 0.03), *PGD* (2.1-fold; *p* = 0.002), and *PFKFB3* (4.3-fold; *p* = 0.003) were significantly reduced in pathways downregulated by high glucose treatment (Table 5).

**Table 5:** Confirmation of mRNA expression of genes from molecular pathways

Genes	Ctrl	+Glc	*p* value	Significance
Group1				
*SRP14*	1.008 ± 0.046	1.419 ± 0.102	0.002	Yes
*SRP54*	1.038 ± 0.101	1.804 ± 0.113	0.0003	Yes
*SRP72*	1.095 ± 0.20	1.177 ± 0.101	0.7509	NS
Group 2				
*UBE2L6*	1.002 ± 0.151	2.034 ± 0,185	0.0007	Yes
*UBE2Q2*	1.000 ± 0.136	1.578 ± 0.139	0.01	Yes
*SKP1*	1.005 ± 0.167	1.516 ± 0.151	0.03	Yes
Group 3				
*ALDOA*	1.000 ± 0.087	0.6652 ± 0.042	0.006	Yes
*ALDH2*	1.000 ± 0.138	0.3593 ± 0.146	0.01	Yes
*PFKP*	1.000 ± 0.088	0.3360 ± 0.074	0.0002	Yes
*PGAM1*	1.000 ± 0,108	0.5777 ± 0.138	0.03	Yes
*PGD*	1.000 ± 0.101	0.4760 ± 0.066	0.002	Yes
*PFKFB3*	1.000 ± 0.179	0.2334 ± 0,082	0.003	Yes

The gene expression levels from molecular pathways were analyzed by RT-qPCR method. The relative changes of mRNA expression levels in the protein export (group1) and ubiquitin-mediated proteolysis (group2) pathways were indicated in the table. The mRNA expression of common genes from molecular pathways that were downregulated by high glucose treatment was shown as group 3 in the table. Statistical significance was determined by Student’s *t* test. Values are expressed as the mean ± SD; *n* = 3 independent experiments with three technical replicates

### Investigation of microarray data on glucose-regulated genes

We also searched our gene expression array data to find relevant candidate gene(s) that may be important in enterocyte glucose metabolism under high-glucose conditions. High glucose significantly induced levels of transcription of *TXNIP* (3.2-fold; *p* = 0.0001) and *LCN15* (6-fold; *p* = 0.0016) genes whereas mRNA levels of *ABCA1* (5.3-fold; *p* = 0.0004) and *IRX3* (13-fold; *p* = 0.0001) were downregulated in the high glucose treatment group (Table 6).

**Table 6:** Glucose-regulated mRNA levels of individual genes

Genes	Ctrl	+Glc	*p* value	Significance
*TXNIP*	1.000 ± 0.140	3.170 ± 0.146	0.0001	Yes
*LCN15*	1.000 ± 0.129	5.992 ± 1.270	0.0016	Yes
*ABCA1*	1.000 ± 0.145	0.1939 ± 0.045	0.0004	Yes
*IRX3*	1.000 ± 0.153	0.07723 ± 0.027	0.0001	Yes

Microarray data indicated individual candidate genes that might be important for intestinal glucose metabolism during high glucose consumption. High glucose treatment significantly induced *TXNIP* and *LCN15* mRNA expression levels relative to the control group while *ABCA1* and *IRX3* mRNA levels were downregulated in the high glucose-treated group. Statistical significance was determined by Student’s *t* test. Values are expressed as the mean ± SD; *n* = 3 independent experiments with three technical replicates

## Discussion

In this study, we used the established in vitro Caco-2 monolayer system to model the effects of high glucose exposure on intestinal enterocytes. The results of TEER measurement and evaluation of the expression of genes encoding the tight junction proteins, OCLN and ZO1, indicated that the cells successfully formed a monolayer barrier that was not disrupted by exposure to high glucose levels.

Initial experiments to evaluate the effects of glucose on known glucose-regulated genes indicated that *SGLT1*, *GLUT5*, and *GLUT2* were downregulated in response to high glucose exposure. The regulations of *SGLT1* and *GLUT5* genes in this paper are in contrast with Mahraoui et al. [17]. They reported that the glucose consumption of early (p29) or late (p198) passages of Caco-2 cells was different, and the mRNA regulations of *SGLT1*, *GLUT5*, and *GLUT2* genes were affected by passage number of the cells and basal glucose consumption rate. Northern blot analysis from the same study indicated that 21-day high glucose (25 mM) treatment of Caco-2 cells can significantly induce *SGLT1* and *GLUT5* mRNA expression levels compared to low glucose-treated groups (1 mM). However, *GLUT2* mRNA level was reduced by high glucose treatment, which we observed similar results for *GLUT2* mRNA expression. There are different steps of experimental approaches between these two studies. We used polarized Caco-2 cells from 35 to 45 passages and maintained Caco-2 cells under high-glucose condition for longer time periods. Moreover, we compared mRNA expression results from 25 and 5.5 mM glucose treatment groups. It seems possible that these factors might affect the regulation of *SGLT1* and *GLUT5* genes. It has been shown that *SGLT1* and *GLUT2* mRNA expression levels were induced when glucose level was increased into the lumen of the intestine in in vivo [18, 19]. It has been indicated that intracellular and extracellular factors are involved in the regulations of glucose transporters [20, 21]. The different mRNA regulations of *SGLT1* and *GLUT2* in in vitro and in in vivo might be due to those factors that are not predominantly regulated in in vitro models under high glucose treatment. Despite these differences, we may at least conclude that high glucose influences the genetic responses of Caco-2 cells.

Several molecular pathways, including intracellular protein export and ubiquitin-mediated proteolysis (upregulated), along with glycolysis/gluconeogenesis, pentose phosphate, and fructose-mannose metabolism (downregulated), were identified as responsive to high glucose in our in vitro enterocyte model. Changes in these molecular pathways under high-glucose conditions should also be evaluated at the functional level to determine the physiological relevance of the findings of the current study to enterocyte glucose metabolism during high glucose treatment in vivo.

A search for genes of particular interest among those identified as regulated by elevated glucose using genome-wide microarray analysis detected several strong candidates, including *ABCA1*, *IRX3*, *TNXIP*, and *LCN15*. ABCA1 is involved in cellular cholesterol efflux and provides cholesterol to high-density lipoproteins (HDL). The intestine expresses high levels of ABCA1, indicating that this tissue is a major cholesterol source for plasma HDL; indeed, intestinal ABCA1 contributes 30% of circulating HDL cholesterol [22]. High dietary cholesterol intake in the intestine increases basolateral ABCA1 mRNA and protein levels in enterocytes [23]. However, high glucose reduces *ABCA1* mRNA and protein levels in the macrophages, decreasing cholesterol flux from the macrophages to the blood; hence, glucose influences cholesterol metabolism via control of *ABCA1* gene expression. Our results also demonstrate that high glucose can reduce *ABCA1* mRNA expression levels in intestinal enterocytes, and levels of cholesterol efflux from the cells in our experimental model should be measured to allow definitive conclusions to be drawn from our results. Surprisingly, *IRX* mRNA expression was also regulated in Caco-2 cells under high glucose treatment, since *IRX3* is highly expressed in the brain and involved in feeding behavior [24]. Single nucleotide polymorphisms in the *IRX3* gene are reported as strongly associated with BMI in human subjects [24]. Moreover, *IRX3* knockout mice had body weights approximately 30% less than their wild-type littermates [24], suggesting that the *IRX3* gene is closely related to obesity. Glucose-regulated *IRX3* mRNA regulation was shown, for the first time in our study; however, the function of intestinal *IRX3* is unknown, and regulation of *IRX3* in the intestine under high glucose consumption requires further investigation. High glucose significantly induced mRNA levels of the *TXNIP* and *LCN15* genes. *TXNIP* plays a role in glucose uptake and is involved in glucose production in the mouse liver [25, 26]. *TXNIP*-mutant mice become hypertriglyceridemic and hypoglycemic during fasting [27]. These observations suggest that *TXNIP* may be an important candidate intracellular regulator of enterocyte glucose metabolism. There is very limited information about *LCN15* in the literature, and its physiological function is unknown; however, it is a member of the lipocalin gene family, of which *LCN2* is a well-studied member involved in obesity and diabetes. *LCN2*, also known as an adipokine, is secreted from the adipose tissue, and its expression is elevated in obese humans and T2DM mouse models [28]. *LCN2*-null male mice are protected from high fat diet-induced obesity and insulin resistance [29]. It was concluded that *LCN2* may be an adipose tissue regulator that modulates glucose metabolism in murine tissues and in the 3T3-L1 adipocyte cell culture model [30]. These studies clearly suggest that *ABCA1*, *IRX3*, *TXNIP*, and *LCN15* may physiologically interact with high glucose in enterocytes. An important finding of the current study is that intestinal glucose absorption may be controlled via *TXNIP* and *LCN15*.

## Conclusions

An important limitation of this study was the use of the colon carcinoma cell line, Caco-2, as a model of the human intestine. Although Caco-2 is a cancer cell line, it is established in the literature as an in vitro model of the human intestine system, and there are no alternative non-cancerous human cell lines that function similarly. The scope of this study was limited to enterocyte glucose metabolism gene regulation during high glucose treatment; therefore, the study findings should be interpreted with caution. However, the results provide valuable information regarding the molecular and genetic responses of enterocytes to high glucose levels, which is the main nutritional contributor to the development of obesity and T2DM. Studies of the intestine and obesity have largely focused on intestinal glucose sensing and reduced intestinal glucose absorption through delaying the breakdown of carbohydrates in enterocytes, while the mechanisms by which enterocytes respond to high glucose levels have not been investigated previously. Thus, the identification of candidate genes (for example, *TXNIP*) involved in the control of intestinal glucose absorption during high glucose consumption is important. Moreover, enterocytes exposed to high glucose may communicate, possibly via *LCN15*, with endocrine cells in the intestine or peripheral tissues. This screen of glucose-dependent genetic regulation in enterocytes has identified important target genes and will facilitate further investigation of the control of glucose metabolism in enterocytes during high glucose intake, which is a common cause of the progression of obesity and T2DM.

## Methods

### Culture of Caco-2 cells

The human colorectal adenocarcinoma epithelial cell line, Caco-2, was purchased from the American Type Culture Collection (ATCC, HTB-37). Caco-2 cells were maintained at 37 °C in a 5% CO_2_/95% O_2_ atmosphere. They were grown in DMEM supplemented with 15% (*v*:*v*) fetal bovine serum (Gibco, Cat. No.: 10500), 1% penicillin and streptomycin solution with antimycotic (Sigma, Cat. No.: A5955), and 1% (*v*:*v*) nonessential amino acids (Gibco, Cat. No.: 11140). Cells were separated into two groups: control (ctrl) and high glucose-treated (+Glc), which were grown in DMEM cell culture medium containing different glucose concentrations (selected based on a previous report [31]) as follows: ctrl group 5.5 mM glucose (Sigma, Cat. No.: D6046) and +Glc group 25 mM glucose (Sigma, Cat. No.: D6429). Cells were maintained in 5.5 and 25 mM DMEM cell culture media for three passages (28 days) to acclimate to the different glucose conditions. The experiments were performed when Caco-2 cells were between passages 35 and 45.

### Modeling of the human intestinal system and glucose treatment

When Caco-2 cells are grown in a bicameral cell culture system, they can polarize to produce apical and basolateral surfaces [32], mimicking the human small intestine [33, 34]. Caco-2 cells (10^6^/cm^2^) from the control and high glucose-treated groups were seeded on collagen-coated polytetrafluoroethylene membrane with 0.4-μm pore size and 1.12 cm^2^ diameter (Corning, Cat. No.: 3493). After 3 days of confluent culture, the cells were grown for an additional 21 days in the bicameral cell culture system in the indicated DMEM media. Media were changed every 2 days during the 21-day experimental period. Transepithelial electrical resistance (TEER) was measured using an Epithelial Volt/Ohm Meter (EVOM; World Precision Instruments, USA) to confirm polarization of the monolayer and cell integrity. The TEER value of polarized Caco-2 cells should be at least 250 Ω/cm^2^ [34].

### Measurement of cellular glucose efflux

We measured glucose efflux to the basolateral surface of the Caco-2 cell monolayer to test the function of the small intestine model. After ctrl and +Glc Caco-2 cells had been cultured for 21 days, the apical surfaces of the polarized cell monolayers were maintained in DMEM containing 5.5 and 25 mM (modeling high dietary glucose consumption) glucose; the basolateral surfaces of both experimental groups were incubated in DMEM containing 5.5 mM glucose. Samples were collected from the basolateral surfaces of the two experimental groups at 0-, 30-, 60-, 120-, 180-, and 240-min time points. The glucose levels on the basolateral surfaces of the monolayers were measured using a Glucose Oxidase Enzymatic Assay kit, according to the manufacturer’s protocol (Sigma, Cat. No.: GAGO20). Data were normalized to total protein levels. Total proteins were extracted from cells using RIPA buffer [35] and their concentrations determined using a BCA protein assay kit (Pierce, Cat. No.: 23227).

### RNA isolation and RT-qPCR

Total RNA was isolated using RNAzol reagent (MRC, Cat. No.: RN190), according to the manufacturer’s protocol. Total RNA samples were dissolved in DEPC water, followed by measurement of their concentrations using a NanoDrop instrument (Thermofisher, MA, USA). Each RNA sample was separated into two aliquots for microarray analysis and RT-qPCR. One microgram of each RNA sample was converted to cDNA using a cDNA synthesis kit (Lifetech, Cat. No.: 4368814). To determine the mRNA expression levels of genes, RT-qPCR was performed on an ABI StepOnePlus instrument (Lifetech, CA, USA) by SYBR-Green mix (Lifetech, Cat. No.: 4367659). The running method of RT-qPCR is as follows: 95 °C/10 min, 40 cycles (95 °C/10 s, 60 °C/1 min), and melt curve stage (95 °C/15 s, 60 °C/1 min). Human cyclophilin A was used as a housekeeping gene for normalization of mRNA expression results. Primer sequences are listed in Additional file 1: Table S1. The 2^−∆∆Ct^ analysis method was applied to calculate mean fold changes in mRNA levels [36].

### Genome-wide microarray analysis

The integrity of RNA samples from each group was assessed using an Agilent 2100 Bioanalyzer and RNA 6000 Nano reagent (Agilent). RNA samples were converted to cRNA using an Ambion Illumina Total Prep RNA Amplification Kit. cRNA samples were loaded onto a microarray (HumanHT-12 v4 Expression BeadChip) then left overnight for hybridization. Finally, the BeadChip was read and analyzed using a microarray laser reader. The significance of the mRNA expression between the control and the high glucose-treated groups was calculated Log_2_ fold change of ± 1.5 with *p < 0.05.*

### Gene ontology and statistical analyses

Significantly regulated mRNAs between the control and the high glucose-treated groups were conducted to DAVID Functional Annotation Bioinformatics Microarray Analysis 6.7 (National Institute of Allergy and Infectious Diseases), to determine statistically overrepresented molecular pathways. KEGG Pathways database was used for functional annotation enrichment analysis. Fisher exact test was used with selected value (*p* ≤ 0.05) in DAVID annotation system. All the results were expressed as means ± SD. Student *t* test was performed for relative gene expression to compare the two groups. GraphPad Prism (version 6.0 for Windows, GraphPad. CA, USA) was used to calculate significance between groups.

## Additional files

Additional file 1: **Table S1.** The Primer list for RT-qPCR. (PDF 856 kb)

Additional file 2: **Table S2.** The list of the significantly downregulated genes under high glucose treatment condition (382 genes). (XLSX 89 kb)

Additional file 3: **Table S3.** The list of the significantly upregulated genes under high glucose treatment condition (297 genes). (XLSX 85 kb)

Additional file 4: **Figure S1.** Ubiquitin mediated proteolysis Pathway (Upregulated). (TIFF 497 kb)

Additional file 5: **Figure S2.** Intracellular protein export pathway (Upregulated). (TIFF 872 kb)

Additional file 6: **Figure S3.** Glycolysis/Gluconeogenesis Pathway (Downregulated). (TIFF 416 kb)

Additional file 7: **Figure S4.** Pentose Phosphate Pathway (Downregulated). (TIFF 490 kb)

Additional file 8: **Figure S5.** Fructose and Mannose Metabolism Pathway (Downregulated). (TIFF 857 kb)

## Abbreviations

ABCA1: ATP-binding cassette, sub-family A member1
ALDH2: Aldehyde dehydrogenase 2
ALDOA: Aldolase A
Caco-2: Colorectal adenocarcinoma epithelial cell
GLUT2: Glucose transporter 2
GLUT5: Glucose transporter 5
IRX3: Iroquois homeobox 3
LCN15: Lipocalin 15
PFKFB3: 6-Phosphofructo-2-kinase/fructose-2,6-biphosphatase 3
PFKP: Phosphofructokinase platelet
PGAM1: Phosphoglycerate mutase 1
PGD: Phosphogluconate dehydrogenase
PTFE: Polytetrafluoroethylene
SGLT1: Sodium-dependent glucose transporter 1
SKP1: S-phase kinase-associated protein 1
SRP14/54/72: Signal recognition particle 14 kDa/54 kDa/72 kDa
T2DM: Type 2 diabetes mellitus
TEER: Transepithelial electrical resistance
TXNIP: Thioredoxin-interacting protein
UBE2L6: Ubiquitin-conjugating enzyme *E2L 6*
UBE2Q2: Ubiquitin-conjugating enzyme E2Q family member 2
