# Supplementary material for: Investigation of the influence of high glucose on molecular and genetic responses: an in vitro study using a human intestine model
Source: Genes Nutr. 2018 Apr 30;13:11. doi: 10.1186/s12263-018-0602-x (PMC5928582; doi:10.1186/s12263-018-0602-x)
Supplement: Supplementary file 1 — Table S1. The Primer list for RT-qPCR. (PDF 856 kb) [file 12263_2018_602_MOESM1_ESM.pdf]

Table S1

**Gene Symbol**	**Forward (5'-3')**	**Reverse (5'-3')**	**Reference (PMID)**
**Abca1**	GCACTGAGGAAGATGCTGAAA	AGTTCCTGGAAGGTCTTGTTCAC	11719471
**Aldoa**	TCACCGCATCGTGGCACCTG	GAAGCGCCGGTTCTCCTCGG	25932951
**Aldh2**	CACTTCGCCCTGTTCTTCAACC	CCTGCTCGGTCTTGCTATCAAAG	21593812
**Glut2**	CTCTCCTTGCTCCTCCTCCT	TTGGGAGTCCTGTCAATTCC	25010715
**Glut5**	TCTCCTTGCAAACGTAGATGG	GAAGAAGGGCAGCAGAAGG	24426192
**Irx**	CTCTCCCTGCTGGGCTCT	CAAGGCACTACAGCGATCTG	25512384
**Ocln**	ATGAGACAGACTACACAACTGG	TTGTATTCATCAGCAGCAGC	23668856
**Pfkp**	GCATGGGTATCTACGTGGGG	CTCTGCGATGTTTGAGCCTC	25932951
**Pfkfb3**	CTGGACAGGGAGGGAGATACTA	AATGAAGAGCTTTGCCCGTGGTC	16115917
**Pgam1**	AGGTCACTGCCTACTGCCTG	ACATCACCACGCAGGTTACAT	25932951
**Pgd**	TTATTTGTGGGGAGCGGAGT	TCTTTGTTCCCTCCTGGCAT	26147000
**Sglt1**	TGGCAATCACTGCCCTTTA	TGCAAGGTGTCCGTGTAAAT	24426192
**Txnip**	ACAGAAAAGGATTCTGTGAAGGTGAT	GCCATTGGCAAGGTAAGTGTG	18171713
**Zo1**	GAATGATGGTTGGTATGGTGCG	TCAGAAGTGTGTCTACTGTCCG	23668856
**Cypa**	TACGGGTCCTGGCATCTTG	CGAGTTGTCCACAGTCAGCA	*
**Lcn15**	GCATCTGACTGCAGGGTCTT	CCTTCAGGTACTCGGCATCC	*
**Skp1**	AGGAGATGATGACCCAGTTCCT	GAGGGTCATCCTTGTGGTGG	*
**Srp14**	CGGGCAGCGTCTATATCACC	CTTGGAGCTCACCACAGTGCT	*
**Srp54**	GCGGTGTCTTTTGCGAGTTC	ATCTGCTTCCAACAAAGCGG	*
**Srp72**	TGAGGTCACATGGGTTTGGAC	ATCAATGGGCCAAACAGACCT	*
**Ube2L6**	CCCAGAGCCAAGGGAGTTTA	TGCAAGGTGACCTGTCTCTC	*
**Ube2Q2**	GGGTCAGTGCAAGCTTCAGAT	TCCACCCAATACATACCCTCCT	*
	* Refers that primers were designed by authors		
