# Supplementary figures and images for: Investigation of the influence of high glucose on molecular and genetic responses: an in vitro study using a human intestine model
Source: Genes Nutr. 2018 Apr 30;13:11. doi: 10.1186/s12263-018-0602-x (PMC5928582; doi:10.1186/s12263-018-0602-x)

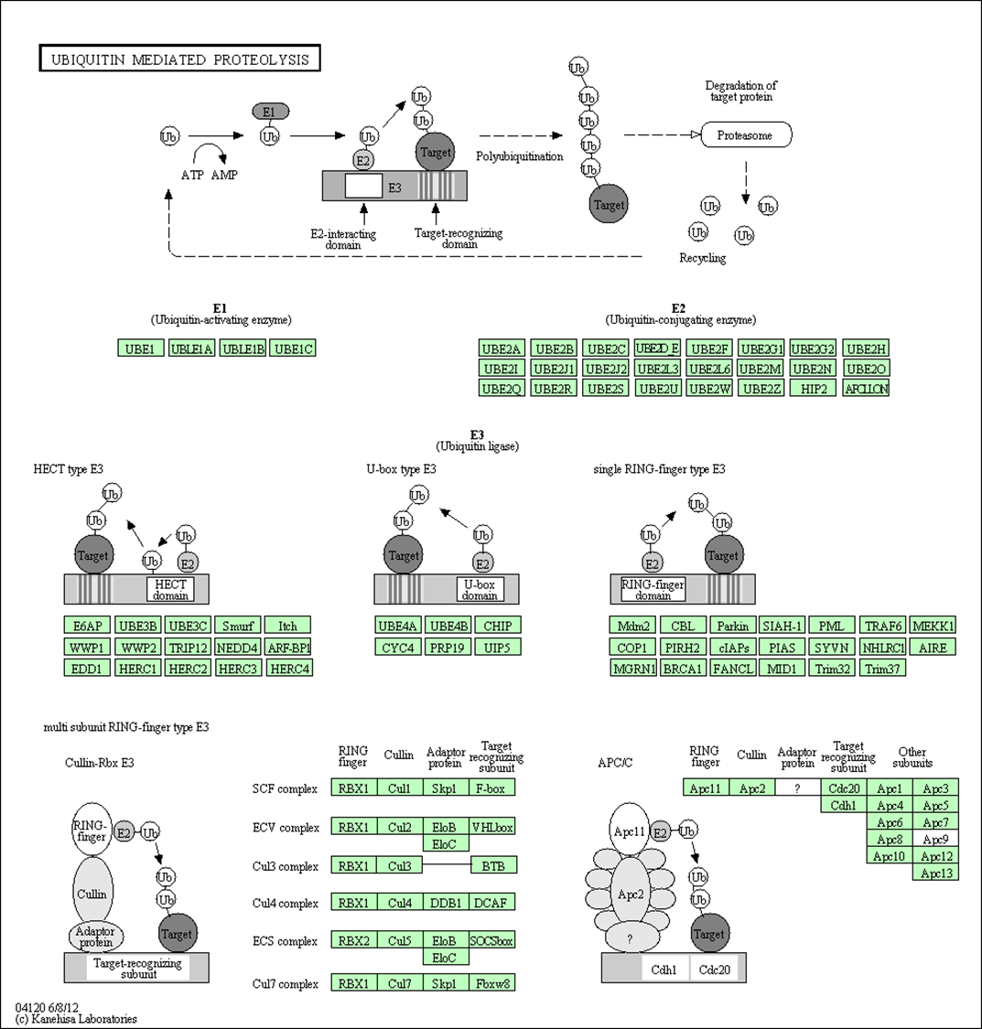

Supplement: Supplementary file 4 — Figure S1. Ubiquitin mediated proteolysis Pathway (Upregulated). (TIFF 497 kb) [file 12263_2018_602_MOESM4_ESM.tif]

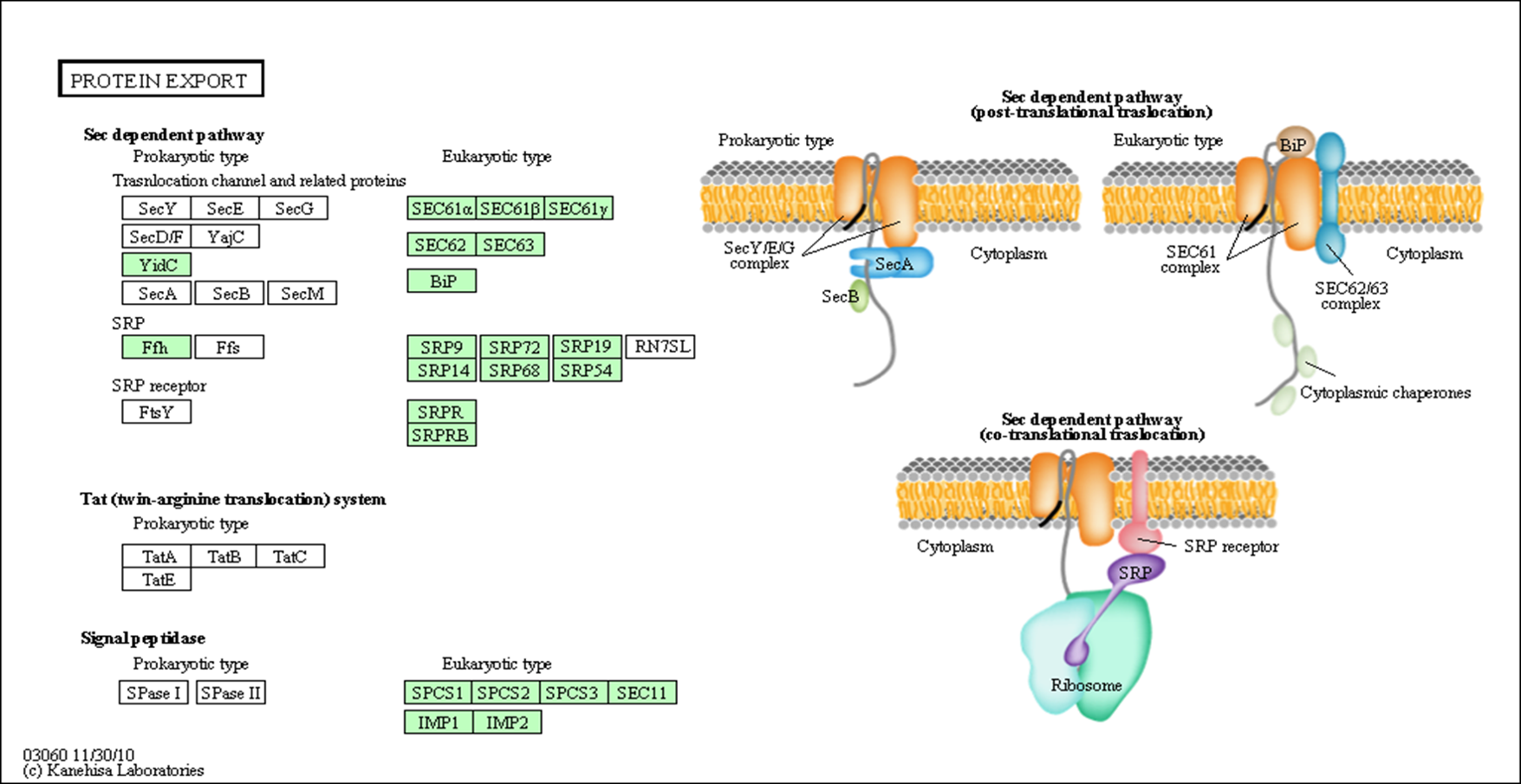

Supplement: Supplementary file 5 — Figure S2. Intracellular protein export pathway (Upregulated). (TIFF 872 kb) [file 12263_2018_602_MOESM5_ESM.tif]

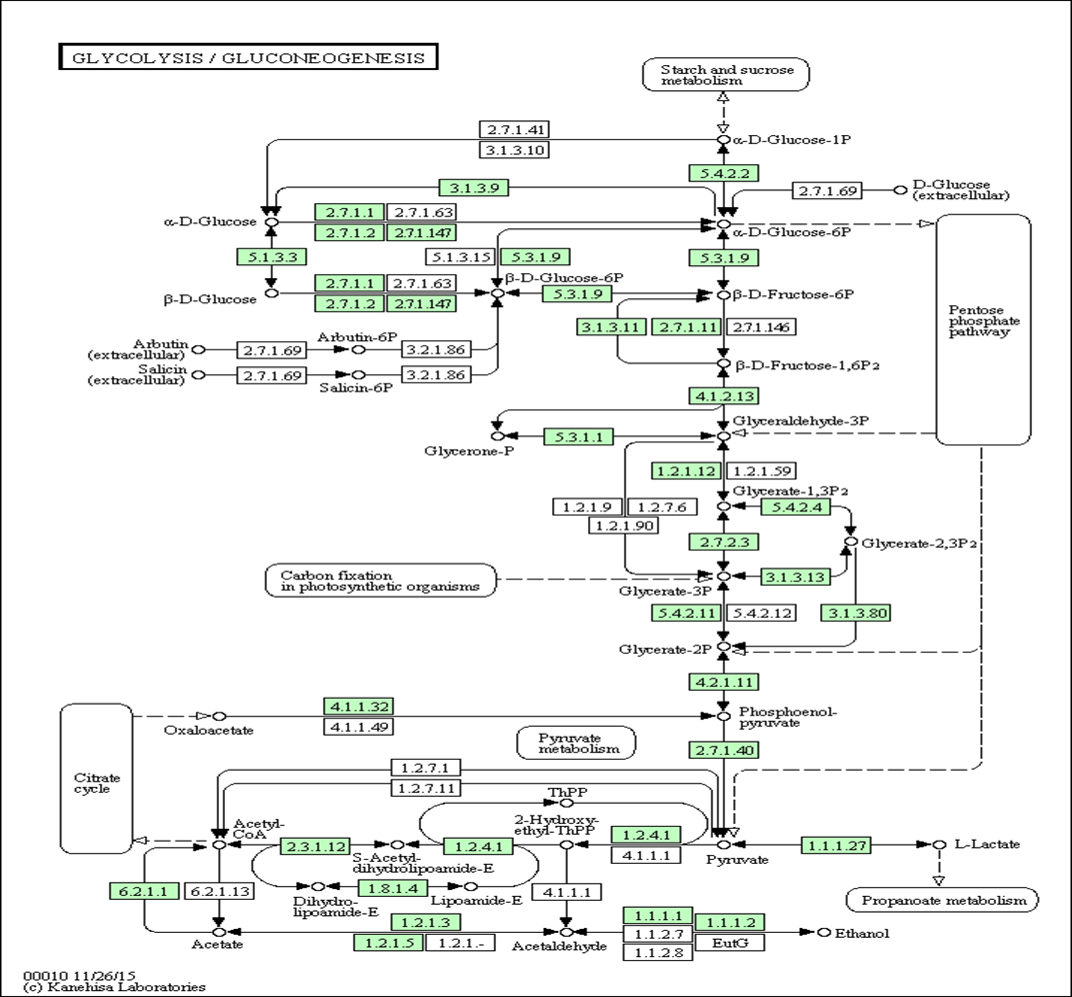

Supplement: Supplementary file 6 — Figure S3. Glycolysis/Gluconeogenesis Pathway (Downregulated). (TIFF 416 kb) [file 12263_2018_602_MOESM6_ESM.tif]

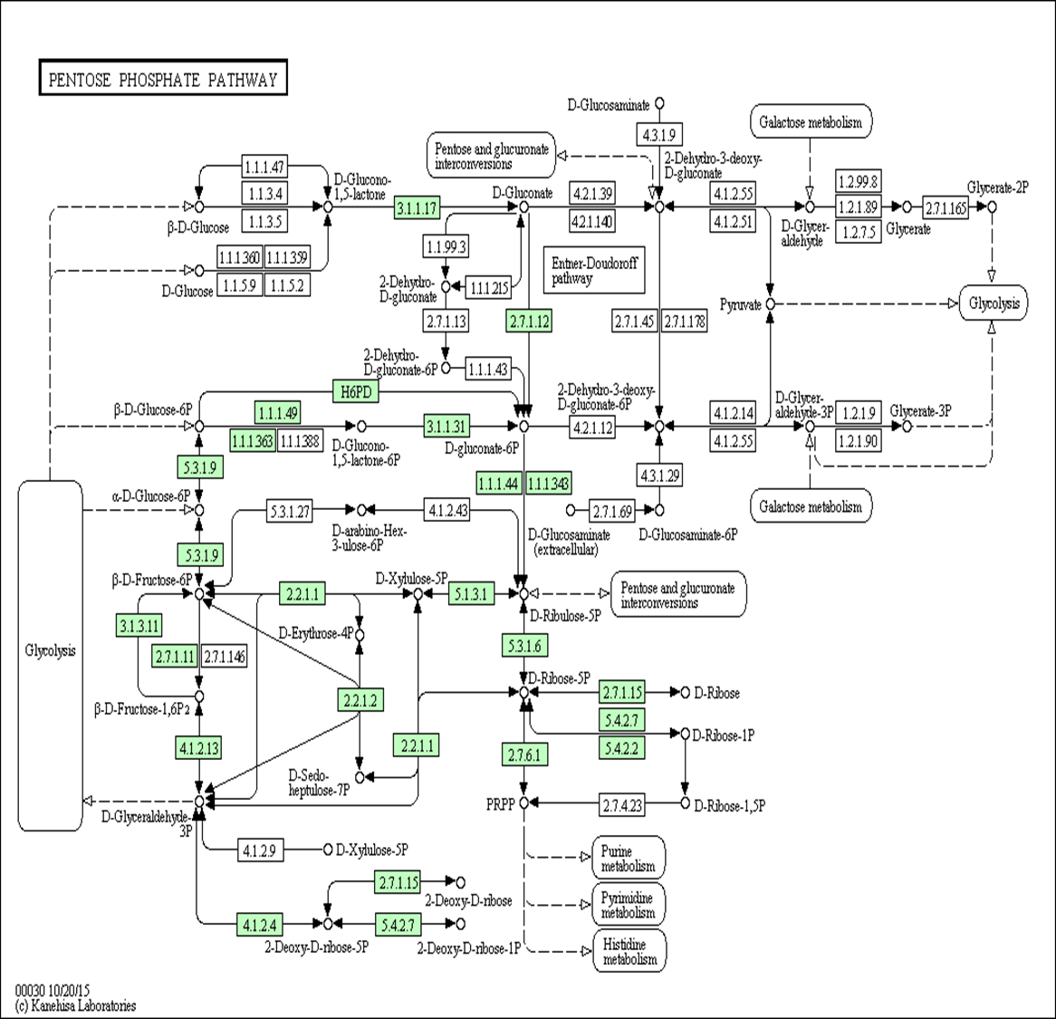

Supplement: Supplementary file 7 — Figure S4. Pentose Phosphate Pathway (Downregulated). (TIFF 490 kb) [file 12263_2018_602_MOESM7_ESM.tif]

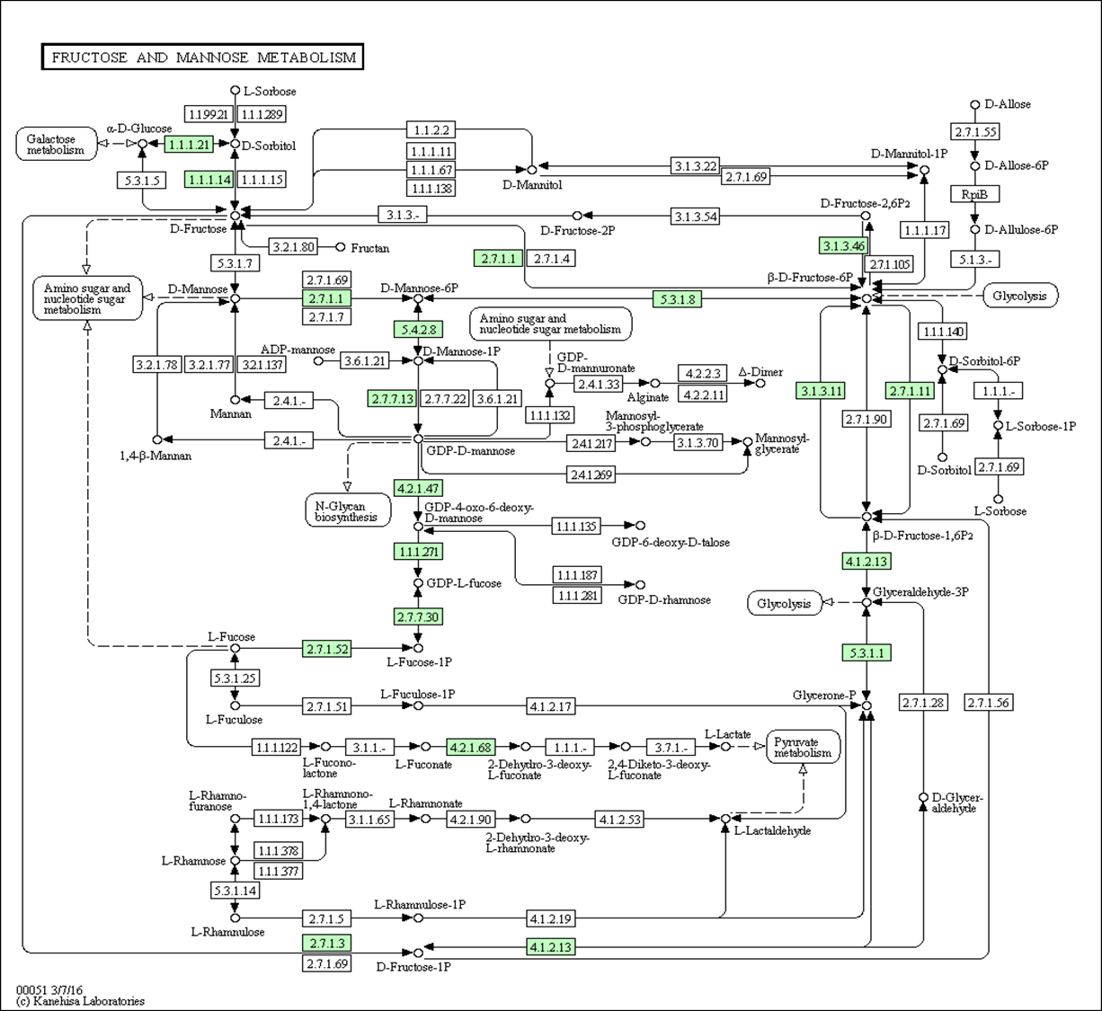

Supplement: Supplementary file 8 — Figure S5. Fructose and Mannose Metabolism Pathway (Downregulated). (TIFF 857 kb) [file 12263_2018_602_MOESM8_ESM.tif]
